# A super-elderly patient with distal biliary tract cancer and tumor-associated granuloma: a case report

**DOI:** 10.3389/fonc.2026.1755032

**Published:** 2026-07-03

**Authors:** Longfei Chen, Rong Ran, Jing Wu, Futang Li, Zikai Wei, Zhiwei He, Chao Yu

**Affiliations:** 1 Department of Hepatobiliary Surgery, The Affiliated Hospital of Guizhou Medical University, Guizhou Medical University, Guiyang, Guizhou, China; 2 College of Clinical Medicine, Guizhou Medical University, Guiyang, Guizhou, China; 3 Guizhou Provincial Institute of Hepatobiliary, Pancreatic and Splenic Diseases, Guiyang, Guizhou, China; 4 Key Laboratory of Liver, Gallbladder, Pancreas and Spleen of Guizhou Medical University, Guiyang, Guizhou, China; 5 Guizhou Provincial Clinical Medical Research Center of Hepatobiliary Surgery, Guiyang, Guizhou, China; 6 Key Laboratory of Hepatobiliary and Pancreatic Diseases Treatment and Bioinformatics Research, Guizhou Medical University, Guiyang, Guizhou, China

**Keywords:** dCCA, minimally invasive surgery, pancreaticoduodenectomy, super-elderly, tumor-associated granulomatous reaction

## Abstract

**Background:**

Distal cholangiocarcinoma (dCCA) has a poor prognosis, and radical surgery is the only potential curative approach. However, elderly patients (≥80 years old) are often excluded from surgery due to high perioperative risks, and related reports are rare.

**Case presentation:**

This article reports a 90-year-old female patient who presented with upper abdominal pain and distension. Imaging studies suggested a mass in the head of the pancreas. After undergoing pancreaticoduodenectomy, the pathology confirmed poorly differentiated cholangiocarcinoma, with negative margins (R0 resection). Notably, no cancer metastasis was found in the regional lymph nodes, but non-caseating granulomas were observed, which were considered to be tumor-related granulomatous reactions.

**Treatment and outcomes:**

The patient received meticulous volume management, anti-infection, and supportive treatment after surgery and was discharged in good condition. Postoperatively, she experienced transient cardiac function stress and secondary fungal infection, which were controlled after symptomatic treatment.

**Conclusion:**

This case indicates that age should not be an absolute contraindication for radical surgery in elderly dCCA patients with acceptable physical conditions after strict evaluation. Through precise surgery and multidisciplinary perioperative management, safe resection and survival benefits can be achieved. At the same time, clinicians need to be vigilant about this rare phenomenon of tumor-related granulomatous reactions and incorporate it into the differential diagnosis and follow-up system.

## Introduction

Cholangiocarcinoma (CCA) comprises a heterogeneous group of malignancies arising from the biliary epithelium and accounts for approximately 3% of all gastrointestinal malignancies (1). On the basis of anatomic origin, it is typically subdivided into intrahepatic (iCCA), perihilar (pCCA), and distal (dCCA) cholangiocarcinoma (2). Most cases are pCCA (60%), whereas dCCA is less common, accounting for about 30% (3). The overall incidence of biliary tract tumors, including dCCA, continues to rise and is positively associated with age; prevalence in older adults is nearly twice that in younger individuals (4, 5). Owing to the limited efficacy of radiotherapy and chemotherapy, definitive surgical resection is the sole therapy with curative potential (6, 7), nevertheless, the 5-year survival in dCCA is approximately 20% (8). However, older adults have been consistently underrepresented in oncology trials, limiting the evidence base that guides clinical decision-making. Given their elevated perioperative risk, surgery in the very elderly was historically avoided and, in many settings, treated as a de facto contraindication.

Tumor-associated granulomatous inflammation is a localized entity characterized by noncaseating granulomas at sites of involvement. When systemic sarcoidosis is clinically absent, noncaseating epithelioid granulomas may occur in regional draining nodes of a malignancy, within the index tumor, or even at extraregional locations (9). Evidence-based clinical management and therapeutic strategies for tumor-associated sarcoid-like reaction remain poorly defined. Its pathogenesis is incompletely understood, and the relationship between sarcoidosis and malignancy remains controversial, as sarcoidosis may arise during the course of cancer and, conversely, malignancy may develop in patients with sarcoidosis (10). In this study, we report a rare case of a 90-year-old Super-elderly Patient with dCCA who underwent surgical management complicated by a tumor-associated sarcoid-like reaction. To our knowledge, this is among the oldest reported cases of distal cholangiocarcinoma treated surgically.

## Case presentation

**Figure 1:**
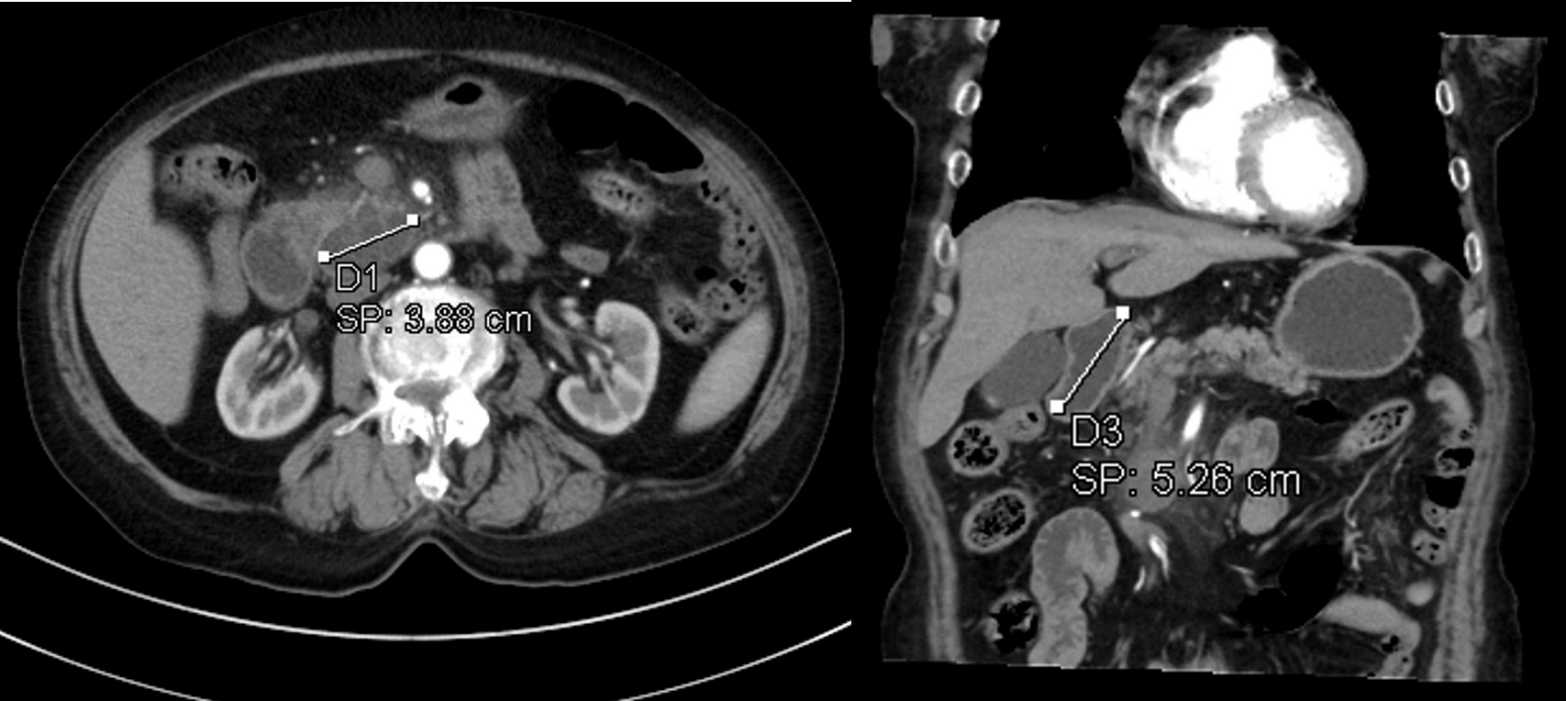
Space-occupying lesion in the uncinate process of the pancreas: Multiple cystic and patchy mixed density shadows are seen in the uncinate process of the pancreas, with unclear boundaries. The largest cross-section is approximately 38 × 52 mm. Multiple septa are present inside, with rough edges and flocculent high-density shadows. The septa and cavity show heterogeneous enhancement.

An outside-hospital CT in a 90-year-old woman showed a pancreatic head mass, concerning for neoplasm. Seeking higher-quality medical care, she presented to our hospital with a 5-day history of epigastric pain accompanied by abdominal distension, worsened after meals. She had no history of cardiovascular disease or other major chronic illnesses. Because of the patient’s extremely advanced age, a detailed preoperative multidisciplinary assessment was performed before surgery, involving hepatobiliary surgeons, anesthesiologists, intensive care specialists, and geriatric medicine specialists. The assessment included general physical condition, daily activity, major comorbidities, cardiopulmonary reserve, anesthetic risk, nutritional status, and operative tolerance. No absolute contraindication to major surgery was identified. Nutritional evaluation showed mildly decreased serum albumin and total protein levels, with albumin of 37.8 g/L (40–55 g/L) and total protein of 60.79 g/L (65–85 g/L). Laboratory tests also showed chloride 109.65 mmol/L (96.00–108.00 mmol/L), calcium 2.07 mmol/L (2.11–2.52 mmol/L), total bilirubin 45.2 μmol/L, direct bilirubin 16.4 μmol/L, indirect bilirubin 28.8 μmol/L, total bile acids 14.80 µmol/L (≤10 µmol/L), and CA19-9 105.20 U/mL (<27 U/mL). Although a formal comprehensive geriatric assessment scale was not retrospectively available, the available clinical data suggested acceptable functional status, no severe malnutrition, and sufficient perioperative tolerance after multidisciplinary evaluation. Contrast-enhanced CT (**Figure 1**) demonstrated a multilocular cystic, mixed-attenuation lesion in the uncinate process with ill-defined margins, measuring approximately 38 × 52 mm on the coronal plane. Multiple internal septations were present, with heterogeneous enhancement of the septa and intracystic contents (noncontrast/arterial/portal venous phases: 14/29/28 HU). In the absence of histopathology, the radiologic impression favored a mucinous cystic neoplasm/cystadenocarcinoma in the uncinate process.

**Figure 2:**
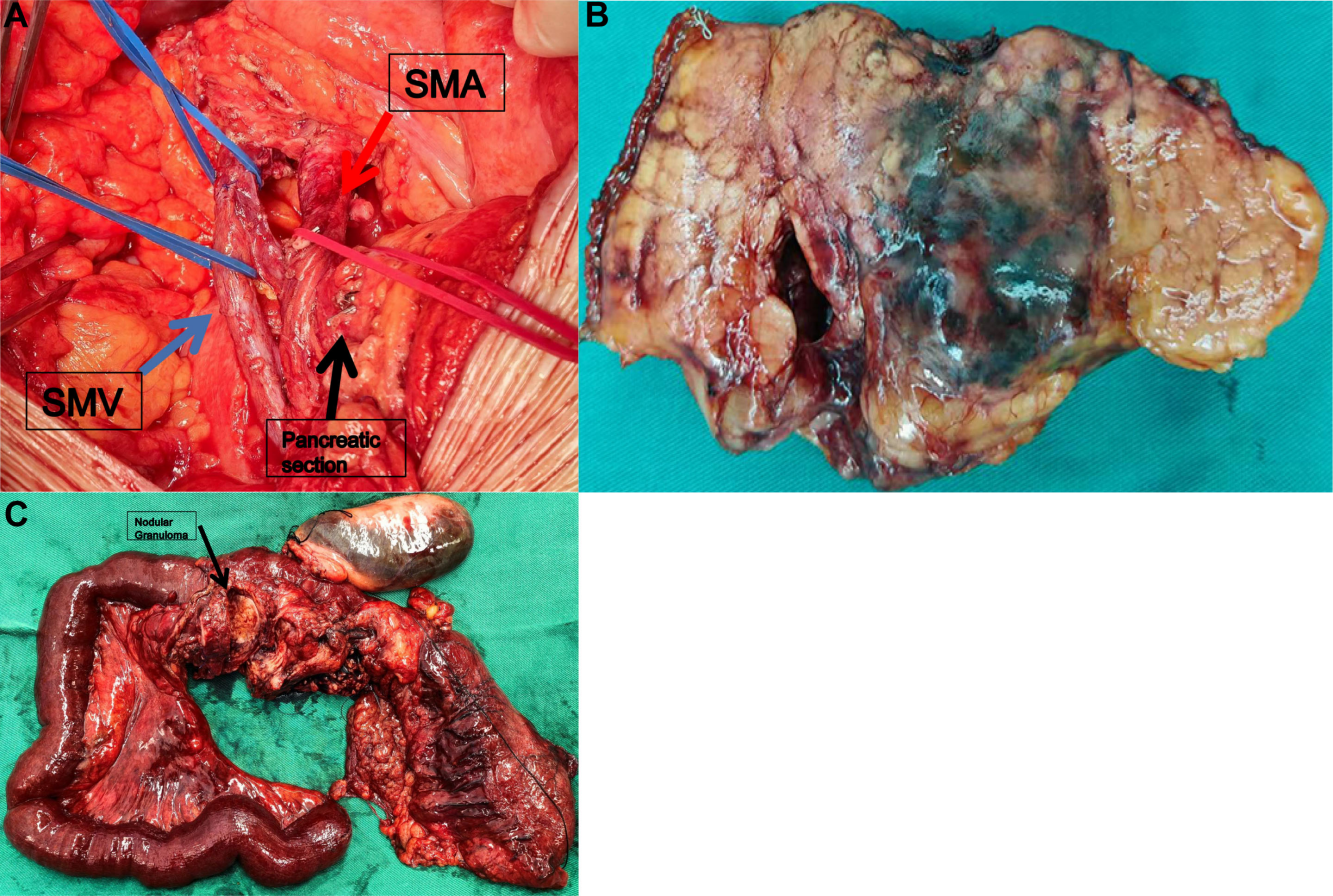
**(A)** Intraoperative anatomy: The superior mesenteric artery (SMA, red arrow), superior mesenteric vein (SMV, blue vessel string traction), and the pancreatic transection surface (black arrow) are exposed, showing the relationship between the tumor and the main blood vessels. **(B)** Specimen of pancreatic uncinate process tumor resection: An irregular mass can be seen, with a surface showing hemorrhagic and necrotic changes. **(C)** Specimen of intestinal and gallbladder resection: The isolated small intestine and gallbladder. Nodular granulomas (arrow) are observed in the intestinal wall.

Although neither EUS nor preoperative tissue sampling was performed, the lesion was considered resectable based on the preoperative imaging findings, and additional endoscopic sampling was not expected to alter the management strategy. In addition, the patient’s family preferred to avoid subjecting her to further invasive procedures that were unlikely to change treatment planning. Despite the patient’s advanced age, preoperative assessment suggested that her general condition was acceptable, with no absolute contraindications to major surgery. Alternative management options, including conservative treatment, palliative biliary drainage, and non-surgical management, were also taken into consideration. However, given the resectability of the lesion, the patient’s overall condition, and the strong preference of the patient and her family for definitive treatment, upfront curative-intent resection was ultimately undertaken. Accordingly, an open pancreaticoduodenectomy (PD) was performed under general anesthesia. Intraoperatively, a firm mass measuring approximately 5 × 5 cm was palpated in the pancreatic head. Because the tumor was located deep in the uncinate process and was in close proximity to the superior mesenteric artery (SMA) and superior mesenteric vein (SMV), an infracolic transmesenteric approach was adopted. The mesocolon was opened in the subcolic region to expose the uncinate process tumor. After meticulous dissection of the surrounding tissues, the SMA and SMV were carefully exposed (**Figure 2A**), both closely adjacent to the lesion. Reconstruction was performed in a Roux-en-Y configuration, in the sequence of pancreaticojejunostomy, hepaticojejunostomy, and gastrojejunostomy. Pancreaticojejunostomy was performed according to the operative record, with an external continuous seromuscular reinforcing layer. For the hepaticojejunostomy, a drainage channel was established at an appropriate distance from the anastomosis. After careful exposure and necessary dissection around the SMA and SMV to define the tumor-vessel interface and facilitate safe resection, the pancreatic head specimen (**Figure 2B**) was removed together with portions of the duodenum and common bile duct. In addition, a 2-cm lesion with a gray-yellow cut surface was identified on the serosal aspect of the intestinal wall (**Figure 2C**). Intraoperative blood loss was only 50 mL. Pathological examination showed no regional lymph node metastasis, and negative resection margins (R0 resection). However, the exact pathological T stage could not be determined because the depth of tumor invasion, which is required for AJCC staging of distal cholangiocarcinoma, was not available. The patient returned to the ward with stable vital signs.

**Figure 3:**
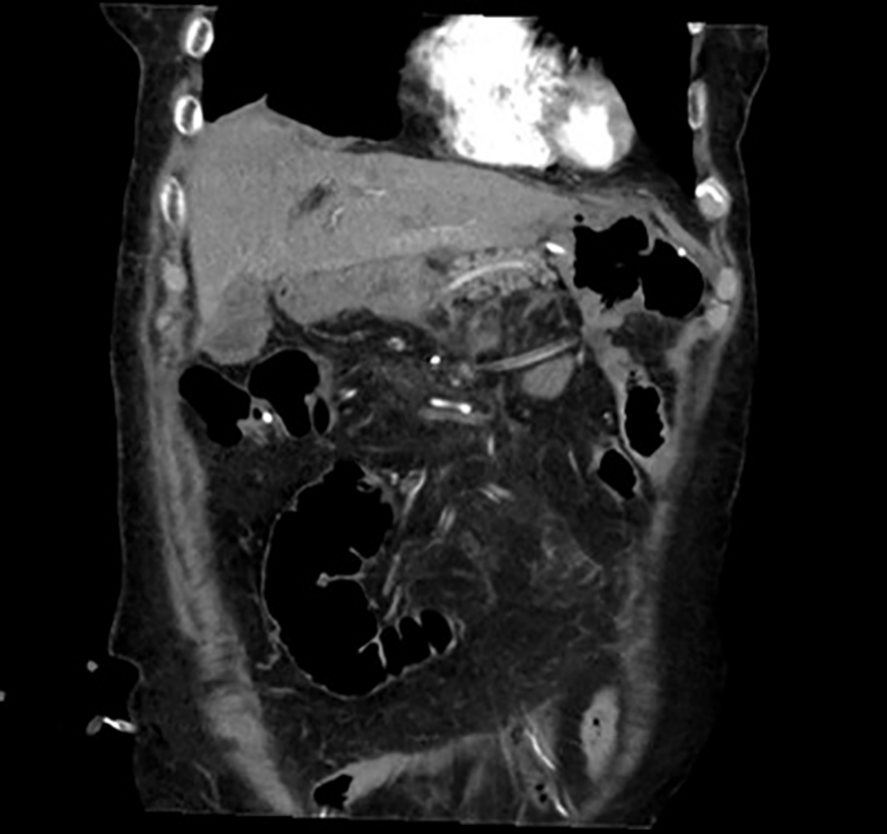
Postoperative imaging examination of the surgical area: A tubular shadow was observed in the pancreatic body and small intestine region, while no such shadow was found in the distal part of the stomach. There was a local anastomosis with the small intestine, and the anastomosis wall was slightly thickened. The enhancement was uniform. The end of the gastric tube was located in the gastric fundus region.

**Figure 4:**
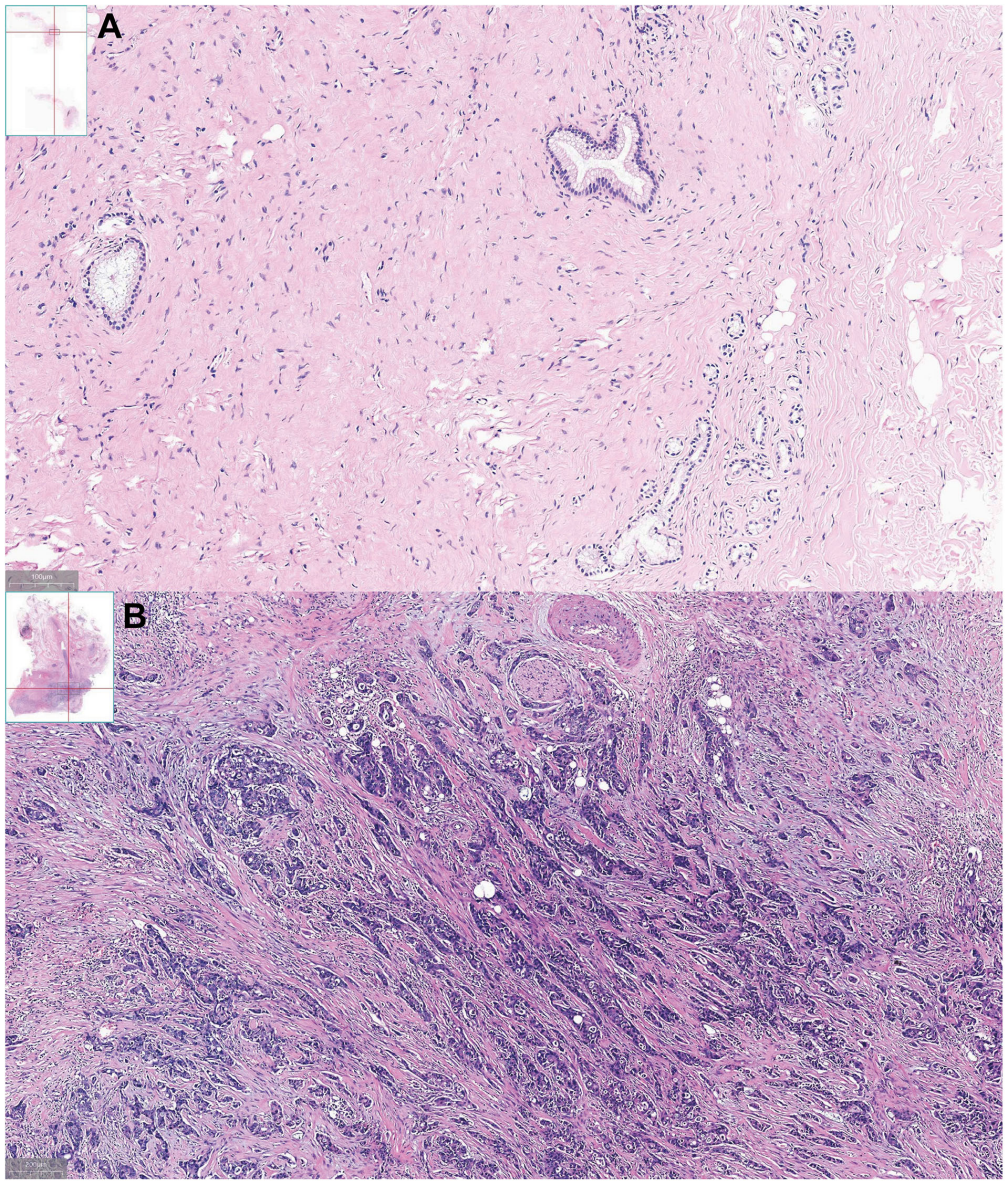
**(A)** Frozen section of the bile duct (HE) during the operation: No tumor tissue was observed. **(B)** Tumor histopathological examination: Tumor pathological result: poorly differentiated cholangiocarcinoma with perineural invasion.

Postoperative imaging showed no hemorrhage or abnormal fluid collection in the operative bed (**Figure 3**). Histopathology revealed a poorly differentiated cholangiocarcinoma (**Figure 4B**) with full-thickness invasion of the bile duct wall and involvement of the duodenal wall, accompanied by extensive perineural invasion. No metastatic carcinoma was identified in 9 lymph nodes from stations 8 and 12 (0/9); granuloma formation was noted within these nodes. Postoperatively, the patient developed transient hypertension and a marked rise in NT-proBNP to 4542 pg/mL, which decreased significantly with diuresis and strict volume management (2391→728 pg/mL), indicating that volume overload/perioperative cardiac stress was the principal contributor.

Postoperatively, the patient’s cardiac stress was managed with diuretic therapy, strict fluid control, and close monitoring, with subsequent clinical improvement. Infection control was initially suboptimal because of prolonged bed rest and concern for postoperative pulmonary infection. Secondary fungal infection was suspected in this context, and antifungal therapy was added after adjustment of the anti-infective regimen. In addition, the patient experienced poor oral intake with nausea and vomiting, as well as a perineal rash related to prolonged bed rest and sleep disturbance. These issues gradually improved with nutritional support and symptomatic treatment. The patient was hospitalized for a total of 8 days, including 5 days after surgery, and was eventually discharged in stable condition. Postoperative oncological surveillance was planned every 3–6 months during the first 2 years after surgery and every 6–12 months thereafter up to 5 years, including clinical assessment, liver function tests, serum tumor marker monitoring, and abdominal imaging. The patient completed follow-up examinations at a local hospital at 3 and 6 months after surgery, and neither imaging examinations nor tumor marker assessments showed evidence of tumor recurrence. Through regular telephone follow-up with the patient’s family, we confirmed that the patient remained alive, had no evidence of disease, was in good general condition, and had no postoperative complications. Compared with younger patients, postoperative complications in the super-elderly are more frequent and heterogeneous; issues that are common or mild in younger individuals can become pivotal in this population. Prompt and appropriate management is key to preventing escalation of complications.

## Discussion

Approximately one-third of incident cancer diagnoses occur in individuals aged >75 years, a share projected to increase in the future (11, 12). Against the backdrop of global population aging, “older adults with cancer” are increasingly being defined as a distinct patient population. Although curative−intent surgical resection remains the optimal therapy for solid tumors—including dCCA—aging introduces pronounced interindividual heterogeneity: declining functional status, worsening nutritional state, diminished organ reserve, multimorbidity, and polypharmacy. These factors lead many clinicians to consider older patients at higher risk for perioperative complications. The central question is whether perioperative risks outweigh the oncologic benefits of curative resection, particularly in vulnerable patients with shortened life expectancy; in this group, carefully balancing the potential surgical benefit against the anticipated survival gain is especially critical. Some investigators have recognized this issue and conducted clinical research analyses focusing on elderly patients with biliary tract cancer. Weigle et al. (13) found no statistically significant difference in the incidence of postoperative complications between the Super-elderly and other patients (27.3% vs 34.3%; p = 0.754). Bartsch et al. reported comparable short−term outcomes by age, with similar resectability (≥70 vs <70: p = 0.188), extent of resection, and perioperative morbidity (≥70 vs <70: p = 0.797) (14). In the multicenter analysis by Vitale et al. (4) including 129 older and 455 younger patients, those aged ≥70 years had a significantly higher complication rate, whereas mortality, progression−free survival (PFS), and overall survival (OS) did not differ significantly between the two groups. Similarly, in a study of ICC, patients in both age groups who underwent surgery—either alone or as part of multimodal therapy—had markedly better outcomes than those treated with chemotherapy alone, radiotherapy alone, or conservative management, indicating a clear survival advantage associated with surgery in older ICC patients (15). Although dCCA and ICC share a common origin and show highly similar patterns of development, their surgical management differs. By contrast, as a periampullary malignancy, dCCA is treated with the same operative approach as PDAC, namely PD. Renz et al. analyzing 300 patients, found that outcomes in patients aged ≥75 years undergoing PD were comparable to those in younger patients, with no differences in perioperative mortality, postoperative complications, or OS (16). Other studies have reported similar findings (17), indicating that age alone should not be a limiting factor for surgical treatment in older patients with cancer. Because there are currently no studies specifically evaluating survival outcomes in Super-elderly patients with dCCA, we can only provide a preliminary, indirect assessment of prognosis by drawing separately on evidence from studies focused on the tumor type and on the surgical approach. Given the high prevalence of medical comorbidities and the biological characteristics of certain tumors, many cancers may be more challenging to treat in older patients (18). Moreover, optimal dosing and accurate prediction of treatment−related toxicity remain challenging. Because clinical trial eligibility criteria often impose an upper age limit of 70 years, older adults with cancer are underrepresented in clinical research (19). Curative−intent oncologic resection can still yield survival benefits in Super−elderly patients. This further supports that surgery should not be abandoned as an active treatment option in this population.

However, from an oncologic perspective, the prognosis in this case should still be interpreted cautiously. The tumor was poorly differentiated and accompanied by perineural invasion, both of which are generally associated with more aggressive biological behavior and a less favorable prognosis. Although R0 resection was achieved and the patient recovered from surgery, the long-term oncologic benefit of surgery cannot be determined from the immediate postoperative course alone. Adjuvant therapy was also considered; however, in view of the patient’s extreme age, expected tolerance, and the balance between potential benefit and treatment-related burden, it was not prioritized. Therefore, the clinical value of surgery in this case lies primarily in achieving definitive resection in a carefully selected nonagenarian patient, while long-term outcome assessment still depends on continued follow-up.

In super-elderly patients, postoperative quality of life should also be considered when deciding on aggressive surgery. Beyond tumor resectability and operative tolerance, recovery of oral intake, mobility, independence in daily living, and the risk of functional decline should be evaluated. Therefore, this case represents individualized decision-making in a carefully selected patient rather than a general recommendation for aggressive surgery in all super-elderly patients.

The absence of preoperative biopsy also requires ethical consideration. In this case, the lesion was considered resectable on imaging, and additional biopsy was unlikely to change the treatment strategy but would have added another invasive procedure for a nonagenarian patient. Conservative treatment, palliative management, and curative-intent surgery were discussed with the patient and her family. The final decision for pancreaticoduodenectomy was made after multidisciplinary evaluation and informed consent, with careful consideration of surgical risk, potential benefit, and patient preference.

Notably, in the absence of imaging and laboratory evidence suggestive of sarcoidosis, an intestinal mass was identified intraoperatively. Postoperative histopathology demonstrated granulomatous inflammation within the lesion, raising suspicion for a metastatic intestinal tuberculous focus; however, chest CT showed no evidence of pulmonary TB lesions. In addition, nucleic acid testing for the Mycobacterium tuberculosis complex performed on lymph node specimens was negative. Given the patient’s denial of a tuberculosis history and the absence of clinical manifestations consistent with intestinal TB, we provisionally consider a rare tumor−associated sarcoid−like granulomatous reaction, which shares the histopathologic features of sarcoidosis but lacks systemic involvement (20). This entity may occur in the tumor stroma of the primary lesion, in draining lymph nodes, and even in distant nonregional tissues, and is pathologically characterized by compact noncaseating epithelioid granulomas with multinucleated giant cells, representing a tumor−related immune reactive change (21–23).

Tumor-associated granulomatous reactions are uncommon but have been described in patients with a variety of hematologic and solid malignancies, including some cases occurring in the setting of immune checkpoint inhibitor therapy (24–28). Although their exact pathogenesis remains unclear, they are generally regarded as immune-mediated lesions involving macrophage and T-cell responses, with cytokines such as tumor necrosis factor contributing to granuloma formation (29). In the context of malignancy, the main clinical significance of this phenomenon lies in avoiding misinterpretation as metastatic disease or tumor recurrence, which may otherwise lead to inaccurate staging and inappropriate management (26). The majority of tumor−associated sarcoid−like granulomatous reactions are identified at the time of primary tumor resection (30–33); however, some are detected several years after surgery, attributable to pharmacologic or non−pharmacologic induction (34–36). Because sarcoid−like granulomatous reactions are difficult to diagnose preoperatively by imaging, the pretest likelihood is typically low. Postoperative examination of resected specimens to confirm granuloma formation can facilitate their recognition. In our case, the granulomatous inflammation observed in the initial specimen should be considered a tumor−associated sarcoid−like reaction. Although such reactions are not associated with overall survival or recurrence−free survival (37) and are not regarded as an indication for therapy, we will nonetheless pursue long−term follow−up.

This case highlights several practical points for surgeons. First, pancreaticoduodenectomy in a nonagenarian patient should not be ruled out solely on the basis of chronological age; rather, patient selection should rely on a comprehensive assessment of general condition, resectability, operative tolerance, and patient preference. Second, the decision threshold in such patients should be individualized and based on a balance between operative risk, the potential benefit of definitive resection, and the limitations of non-surgical alternatives. Third, granulomatous lesions encountered in the setting of malignancy require careful interpretation, as tumor-associated sarcoid-like reactions may mimic nodal metastasis or tumor recurrence and thereby affect staging and management. Accordingly, the main clinical value of this case lies not only in its rarity, but also in illustrating the importance of individualized surgical decision-making and cautious pathological interpretation in Super-elderly patients with resectable periampullary malignancy.

## Summary

In summary, we report a Super−elderly patient with dCCA concomitant with rare tumor−associated sarcoid−like granulomatous nodules. With comprehensive preoperative evaluation, individualized surgical planning, and meticulous perioperative management, Super-elderly dCCA patients can still benefit from curative−intent surgery. When sarcoid−like granulomas are identified intraoperatively or postoperatively, clinicians should heighten recognition and pursue careful differential diagnosis, and incorporate these findings into long−term surveillance frameworks to optimize overall management and prognostic assessment in elderly patients with biliary tract malignancies.

## Data Availability

The raw data supporting the conclusions of this article will be made available by the authors, without undue reservation.

## References

[1] Ilyas SI Khan SA Hallemeier CL Kelley RK Gores GJ . Cholangiocarcinoma - evolving concepts and therapeutic strategies. Nat Rev Clin Oncol. (2018) 15:95–111. doi: 10.1038/nrclinonc.2017.157 28994423 PMC5819599

[2] Ilyas SI Gores GJ . Pathogenesis, diagnosis, and management of cholangiocarcinoma. Gastroenterology. (2013) 145:1215–29. doi: 10.1053/j.gastro.2013.10.013 24140396 PMC3862291

[3] Razumilava N Gores GJ . Cholangiocarcinoma. Lancet. (2014) 383:2168–79. doi: 10.14309/00000434-201710001-02827 24581682 PMC4069226

[4] Vitale A Spolverato G Bagante F Gani F Popescu I Marques HP . A multi-institutional analysis of elderly patients undergoing a liver resection for intrahepatic cholangiocarcinoma. J Surg Oncol. (2016) 113:420–426. doi: 10.1002/jso.24148 27100027

[5] Valle JW Kelley RK Nervi B Oh DY Zhu AX . Biliary tract cancer. Lancet. (2021) 397:428–44. doi: 10.1016/s0140-6736(21)00153-7 33516341

[6] Cillo U Fondevila C Donadon M Gringeri E Mocchegiani F Schlitt HJ . Surgery for cholangiocarcinoma. Liver Int. (2019) 39:143–155. doi: 10.1111/liv.14089 30843343 PMC6563077

[7] Frosio F Mocchegiani F Conte G Della Bona E Vecchi A Nicolini D . Neoadjuvant therapy in the treatment of hilar cholangiocarcinoma: review of the literature. World J Gastrointest Surg. (2019) 11:279–286. doi: 10.4240/wjgs.v11.i6.279 31367275 PMC6658363

[8] Nagino M Hirano S Yoshitomi H Aoki T Uesaka K Unno M . Clinical practice guidelines for the management of biliary tract cancers 2019: the 3rd English edition. J Hepatobiliary Pancreat Sci. (2021) 28:26–54. doi: 10.1002/jhbp.870 33259690

[9] Hachisu Y Koga Y Kasama S Kaira K Uno S Yatomi M . The relationship between tumor development and sarcoidosis in aspects of carcinogenesis before and after the onset of sarcoidosis. Medicina (Kaunas). (2022) 58:768. doi: 10.3390/medicina58060768 35744031 PMC9230813

[10] Apalla Z Kemanetzi C Papageorgiou C Bobos M Manoli M Fotiadou C . Challenges in sarcoidosis and sarcoid-like reactions associated with immune checkpoint inhibitors: a narrative review apropos of a case. Dermatol Ther. (2021) 34:e14618. doi: 10.1111/dth.14618 33263945

[11] DeSantis CE Lin CC Mariotto AB Siegel RL Stein KD Kramer JL . Cancer treatment and survivorship statistics, 2014. CA Cancer J Clin. (2014) 64:252–271. doi: 10.3322/caac.21235 24890451

[12] Maddams J Utley M Møller H . Projections of cancer prevalence in the United Kingdom, 2010-2040. Br J Cancer. (2012) 107:1195–202. doi: 10.1038/bjc.2012.366 22892390 PMC3461160

[13] Weigle CA Beetz O Wiemann BA Tessmer P Störzer S Cammann S . Resection of intrahepatic cholangiocarcinoma in octogenarians: a single-center analysis. Discov Oncol. (2024) 15:224. doi: 10.1007/s12672-024-01065-2 38865024 PMC11169410

[14] Bartsch F Baumgart J Tripke V Hoppe-Lotichius M Heinrich S Lang H . Resection of intrahepatic cholangiocarcinoma in elderly patients - is it reasonable. BMC Surg. (2019) 19:157. doi: 10.1016/j.hpb.2016.03.219 31664988 PMC6819605

[15] Zhu H Ji K Wu W Zhao S Zhou J Zhang C . Describing treatment patterns for elderly patients with intrahepatic cholangiocarcinoma and predicting prognosis by a validated model: a population-based study. J Cancer. (2021) 12:3114–3125. doi: 10.7150/jca.53978 33976721 PMC8100797

[16] Renz BW Khalil PN Mikhailov M Graf S Schiergens TS Niess H . Pancreaticoduodenectomy for adenocarcinoma of the pancreatic head is justified in elderly patients: a retrospective cohort study. Int J Surg. (2016) 28:118–125. doi: 10.1016/j.ijsu.2016.02.064 26906329

[17] Xie D Qian B Yang J Peng X Li Y Hu T . Can elderly patients with pancreatic cancer gain survival advantages through more radical surgeries? A SEER-based analysis. Front Oncol. (2020) 10:598048. doi: 10.3389/fonc.2020.598048 33194764 PMC7660699

[18] Tomás CC Oliveira E Sousa D Uba-Chupel M Furtado G Rocha C . Proceedings of the 3rd IPLeiria's International Health Congress: Leiria, Portugal, 6–7 May 2016. BMC Health Serv Res. (2016) 16(Suppl 3):200. doi: 10.1186/s12913-016-1423-5 27409075 PMC4943498

[19] Yasar K Pehlivanoglu F Sengöz A Sengoz G . Coexistence of advanced age and female gender in diabetics with extrapulmonary tuberculosis: four culture-proven cases. Gend Med. (2011) 8:334–8. doi: 10.1016/j.genm.2011.05.008 21689993

[20] González-Cruz C Bodet D Muñoz-Couselo E García-Patos V . Mediastinal FDG-positive lymph nodes simulating melanoma progression: drug-induced sarcoidosis like/lymphadenopathy related to ipilimumab. BMJ Case Rep. (2021) 14:e237310. doi: 10.1136/bcr-2020-237310 33509865 PMC7845685

[21] Brincker H . Sarcoid reactions in Malignant tumours. Cancer Treat Rev. (1986) 13:147–56. doi: 10.1016/0305-7372(86)90002-2 3536088

[22] Chowdhury FU Sheerin F Bradley KM Gleeson FV . Sarcoid-like reaction to Malignancy on whole-body integrated (18)F-FDG PET/CT: prevalence and disease pattern. Clin Radiol. (2009) 64:675–81. doi: 10.1016/j.crad.2009.03.005 19520211

[23] Imai R Tsuchida Y Jinta T . Sarcoidosis or sarcoid-like reaction with mediastinal lymphadenopathy in patients after breast cancer surgery. Respir Investig. (2023) 61:398–404. doi: 10.1016/j.resinv.2023.03.008 37099890

[24] Rodriguez EF Lipson E Suresh K Cappelli LC Monaco SE Maleki Z . Immune checkpoint blocker-related sarcoid-like granulomatous inflammation: a rare adverse event detected in lymph node aspiration cytology of patients treated for advanced Malignant melanoma. Hum Pathol. (2019) 91:69–76. doi: 10.1016/j.humpath.2019.07.001 31279873

[25] Danlos FX Pagès C Baroudjian B Vercellino L Battistella M Mimoun M . Nivolumab-induced sarcoid-like granulomatous reaction in a patient with advanced melanoma. Chest. (2016) 149:e133–e136. doi: 10.1016/j.chest.2015.10.082 27157227

[26] Tetzlaff MT Nelson KC Diab A Staerkel GA Nagarajan P Torres-Cabala CA . Granulomatous/sarcoid-like lesions associated with checkpoint inhibitors: a marker of therapy response in a subset of melanoma patients. J Immunother Cancer. (2018) 6:14. doi: 10.1186/s40425-018-0323-0 29433571 PMC5810034

[27] Cohen PR Kurzrock R . Sarcoidosis and Malignancy. Clin Dermatol. (2007) 25:326–33. doi: 10.1016/j.clindermatol.2007.03.010 17560310

[28] Beutler BD Cohen PR . Sarcoidosis in melanoma patients: case report and literature review. Cancers (Basel). (2015) 7:1005–21. doi: 10.3390/cancers7020821 26083934 PMC4491696

[29] Timmermans WM van Laar JA van Hagen PM van Zelm MC . Immunopathogenesis of granulomas in chronic autoinflammatory diseases. Clin Transl Immunol. (2016) 5:e118. doi: 10.1038/cti.2016.75 28090320 PMC5192066

[30] Pascual-Camps I Alonso-Orcajo N de la Fuente JP Yilmaz T Cordero-Coma M . Sarcoid-like reaction in a patient with uveitis and underlying cancer. Int Ophthalmol. (2017) 37:1235–8. doi: 10.1007/s10792-016-0379-0 27761762

[31] Shi Y Li J Chen M Liu H Ma D Lin Y . Sarcoidosis-like reaction after neoadjuvant pembrolizumab combined with chemotherapy mimicking disease progression of NSCLC induced encouraging discovery of pathological complete response. Thorac Cancer. (2021) 12:3433–3436. doi: 10.1111/1759-7714.14228 34761878 PMC8671890

[32] Zhao X Yue D Qian J Zhang L Song J Zhang B . Case report: sarcoid-like reactions and tertiary lymphoid structures following dual checkpoint inhibition in a patient with early-stage lung adenocarcinoma. Front Immunol. (2022) 13:794217. doi: 10.3389/fimmu.2022.794217 35173719 PMC8841621

[33] Frohlich M Wang H Sakr L . Sarcoid-like reaction discovered on EBUS-TBNA of intrathoracic lymph nodes during immunotherapy for metastatic melanoma. J Immunother. (2020) 43:75–8. doi: 10.1097/cji.0000000000000298 31567703

[34] Craun JB Banks KP Clemenshaw MN Moren RW . Sarcoidlike reaction of neoplasia causing hypermetabolic thoracic adenopathy in setting of extrathoracic Malignancy: report of two cases and a review of the differential diagnostic considerations. J Nucl Med Technol. (2012) 40:231–5. doi: 10.2967/jnmt.112.1028142. PMID: 23015478

[35] Madden TF Baccei SJ . Diffuse bone marrow sarcoid-like reaction associated with renal cell carcinoma. Skeletal Radiol. (2014) 43:1761–6. doi: 10.1007/s00256-014-1960-0 25052538

[36] Iftikhar A Cheema M Ramachandran P Sahni S . Sarcoid-like reaction associated with renal cell carcinoma - a case report. Respir Med Case Rep. (2019) 27:100847. doi: 10.1016/j.rmcr.2019.100847 31024794 PMC6476811

[37] Huh JY Moon DS Song JW . Sarcoid-like reaction in patients with Malignant tumors: long-term clinical course and outcomes. Front Med (Lausanne). (2022) 9:884386. doi: 10.3389/fmed.2022.884386 36059841 PMC9433121

